# The Tensile Strength of Full-Thickness Skin: A Laboratory Study Prior to Its Use as Reinforcement in Parastomal Hernia Repair

**DOI:** 10.3389/fsurg.2019.00069

**Published:** 2019-12-04

**Authors:** Viktor Holmdahl, Olof Backman, Ulf Gunnarsson, Karin Strigård

**Affiliations:** ^1^Department of Surgical and Perioperative Sciences, Umeå University, Umeå, Sweden; ^2^Sunderby Research Unit, Department of Surgical and Perioperative Sciences, Umeå University, Luleå, Sweden

**Keywords:** full-thickness skin, tensile strength, parastomal hernia, composite mesh, biological mesh

## Abstract

**Purpose:** Parastomal hernia is a common complication of an enterostoma. Current methods of repair have high recurrence rates and are associated with severe complications. Autologous full-thickness skin as reinforcement may reduce the recurrence and complication rates. This study aims to investigates the tensile strength of full-thickness skin; information that is essential if we are to proceed with clinical trials on humans.

**Methods:** Full-thickness skin samples from 12 donors were tested for tensile strength, as well as the load tolerated by a suture through the skin. Strips of skin were cut out and stretched until breaking point. Sutures were made through skin samples and traction applied until either the tissue or the suture gave way. All done while recording the forces applied using a dynamometer. Identical tests were carried out on commercially available synthetic and biologic graft material for comparison.

**Results:** The full-thickness skin strips had a median tensile strength of 604 N/cm. This tensile strength was significantly higher than that of the compared materials evaluated in this study. In full-thickness skin, the suture, or tissue endured a median force of 67 N before giving way, which was as high as, or higher than similar sutures through the compared materials.

**Conclusions:** The tensile strength of full-thickness skin vastly exceeds the physiological forces affecting the abdominal wall, and sutures through skin endure high loads before giving way. The tensile strength of a full-thickness skin graft and the strength of sutures through this material will not limit its use for reinforcement in parastomal hernia repair.

## Introduction

In Sweden more than 3,000 patients are operated with a stoma (including urostomy) each year, the majority of which become permanent (1). One of the most prevalent complications of a stoma is parastomal herniation/bulging around the stoma (2–4). Incidence rates vary considerably, in some studies reaching 78% (3–6).

A parastomal hernia differs from other hernias since the stoma itself is an iatrogenic aperture created in the abdominal wall. If the aperture widens more than the diameter of the deviated bowel segment, a hernia inevitably develops.

Relocation of the stoma and primary repair of the defect were methods formerly used for repair but have now been abandoned due to unacceptably high recurrence rates (7, 8). As with inguinal and incisional hernia, the use of mesh has also become standard in the repair of parastomal hernia. However, repair is still associated with high recurrence rates, in some studies up to 46% (8–10). Furthermore, the use of foreign material can lead to serious complications including mesh infection, fistula formation, and erosion of the intestinal wall. These problems have made the safe and reliable repair of parastomal hernia a major surgical challenge that remains to be surmounted.

The use of autologous skin as reinforcement in hernia repairs is an alternative method that may lead to lower recurrence and complication rates by avoiding the use of foreign material. Autologous skin graft has been used in hernia repair before, but to our knowledge only in the onlay position (11–14). Before executing clinical trials on parastomal hernia repair with autologous skin in an intraperitoneal position, experiments on both tissue biology as well as mechanical studies must therefore be performed.

Animal studies at our research center, where autologous full-thickness skin grafts have been placed intraperitoneally have shown promising results, with good graft survival and only minor problems with bowel adhesion (15).

In animal studies, Kama et al. reported the favorable mechanical properties of autologous full-thickness skin grafts implanted in the abdominal wall compared to other repair materials (16). However, more information must be gained regarding the tensile strength of human full-thickness skin.

Our hypothesis in this study was that full-thickness skin has the tensile strength necessary for use as reinforcement in parastomal hernia repair. Our endpoints were: (1) the traction force at the point when breaking of the full-thickness skin strip occurred; (2) the traction force at the point when a suture through full-thickness skin gave way. A secondary hypothesis was that full-thickness skin is at least as strong as commercially available materials currently used in hernia repair.

## Materials and Methods

### Study Design

Full-thickness skin specimens from 14 patients were gathered during surgery where healthy excess skin was removed as part of the procedure. Most of the samples came from the abdominal wall, two came from the breast and one from the gluteal region. Since patients with parastomal hernia range from being young and otherwise healthy to having disseminated cancer and considerable comorbidity, all potential skin donors consenting to take part in this study were included regardless of diagnosis.

Consent was obtained after printed and verbal information had been given. Relevant information on the health of the donors was retrieved from their medical records. This included age, gender, long-term use of corticosteroid medication (>3 months) and relevant disease such as dermatological conditions, cancer, kidney failure, hepatic failure, and diabetes. When the operation to be performed was directly related to the treatment of cancer, this was noted. It was also noted if the patient had received radiation therapy involving the area of skin to be removed. This information was stored in a database, matched to each skin specimen and linked to the tensile strength test results.

The skin samples were immersed in physiological saline directly after removal and tensile strength testing was conducted within a few hours.

Identical tensile strength tests were also performed on Symbotex^TM^ Composite Mesh (Covidien, Dublin, Ireland) and XenMatrix^TM^ Surgical graft (Bard, Murray Hill, NJ, USA) for comparison. Symbotex^TM^ synthetic mesh is manufactured from a 3D textile of monofilament polyester with a pore size of 3.3 mm × 2.3 mm. It has a bioabsorbable porcine-derived collagen film on one side to prevent visceral adhesion. Similar composite meshes are currently used for intraperitoneal reinforcement in ventral and parastomal hernia repair (17, 18). The Xenmatrix^TM^ graft is a biologic reinforcement material composed of an acellular non-crosslinked porcine collagen scaffold, and is commonly used in hernia repair (19, 20).

### Tensile Strength

**Figure 1:**
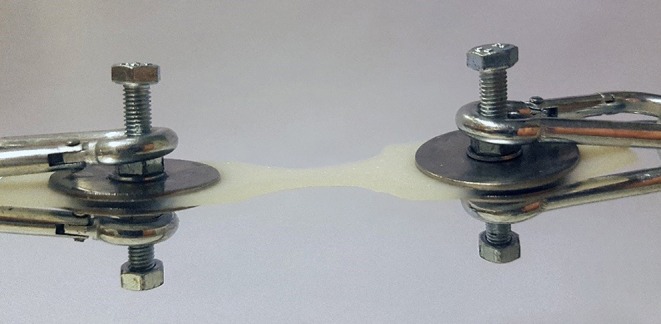
Clamping device with an attached biological collagen matrix sample cut to the dumbbell shape.

**Figure 2:**
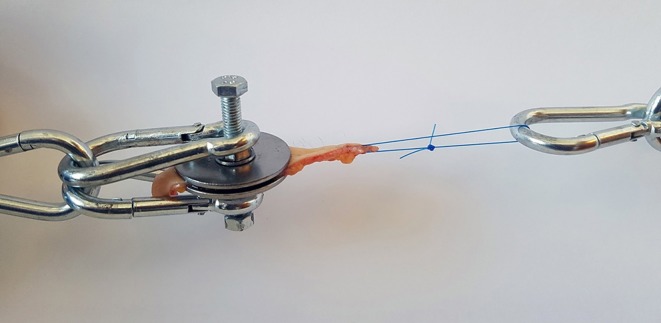
Clamped skin sample with penetrating suture attached to the dynamometer.

Tensile strength was measured using a simple spring-loaded dynamometer capable of traction forces up to 245 N with a precision of 2.5 N. All tests were performed using a specially designed test device. The skin samples were fresh and no chemical preparation was used prior to testing. Subcutaneous fat was sharply dissected from the skin, leaving a specimen with intact dermis, and epidermis. The skin strip used for testing was cut out from the prepared sample in the shape of a dumbbell where the dimensions at the waistline were precisely known. This enabled control of the point of breaking and allowed us to estimate the cross-sectional area at the tearing point. At the points of attachment to the device, two bolts pierced the wider ends of the sample and the tissue was fixed by a nut and washer on either side (Figure 1). During the test, the samples were exposed to a known traction force applied by a spring extended at a speed of 10 mm/min. To investigate the tensile strength of a suture passed through a full-thickness skin sample, tests were performed in a similar fashion on 2–0 and 0 monofilament polypropylene sutures commonly used when closing the linea alba after laparotomy. The skin sample to be tested was fixed at one end of the test device, and the suture passed through the graft at the other end and a loop created with a surgical knot (Figure 2). A traction force was applied to the suture in the same manner as with the skin strip until the tissue or the suture gave way. It was noted whether it was the skin tissue or the suture material that was the first to give way. Identical tests were then performed on the synthetic mesh/graft materials for comparison.

### Statistics

Data on tensile strength were analyzed using the Mann-Whitney *U*-test to detect differences between groups. All statistical calculations were performed using STATA 14 software (StataCorp. College Station, TX, USA).

### Ethics

#### Ethical Approval

This study was approved by the regional board of ethics at Umeå University, Umeå, (reference number: 2016/450-31).

#### Human and Animal Rights

All procedures performed in the study were in accordance with the ethical standards of the institutional research committee and with the 1964 Helsinki declaration and its later amendments or comparable ethical standards. All experiments in the study complied with current law in Sweden. This article does not contain any studies with animals performed by any of the authors.

#### Informed Consent

All patients included had signed a written consent after receiving verbal and printed information. The study is registered at http://www.researchregistry.com; UIN: researchregistry3338.

## Results

All tests were conducted between February and August 2017. The median age of the patients included was 64.5 years ranging from 31 to 82. None of the test subjects suffered from kidney- or hepatic failure. Eight of the patients were operated due to cancer. Patients 4 and 10 had received radiation therapy involving the area of skin to be tested. Summary of patient data may be seen in Table 1. Fourteen skin specimens were obtained. One of the skin samples was lost to the study due to failure of the test equipment, another was lost due to being erroneously placed in formalin after removal at surgery. Two of the skin strips tore between the washers at the point of attachment instead of at the waistline. In these cases, the tensile strength was still calculated based on the width of the waistline and was included in the study. The remaining skin samples broke at the waist. In all, six synthetic mesh and 13 biologic graft samples were tested, all of which broke at the waist.

**Table 1:** Patient characteristics and individual test results.

**Test nr**	**Operation**	**Age-range**	**Cancer**	**Long-term corticosteroid medication**	**Diabetes**	**Thickness**	**Tensile-strength (N/cm)**	**Tensile-strength (N/cm^**2**^)**	**Suture gave way (N)**	**Suture failure 2-0**	**Suture failure** **0**
1	Hemicolectomy	70–80	X	X	X	2, 2	156	713	34	−	0
2	Abdominoplasty	50–60	−	−	−	2, 4	764	3,183	76	0	X
3	Breast reconstruction	60–70	−	−	−	1, 8	396	2,198	83	X	X
4	Breast reconstruction	50–60	−	−	−	2	1,395	6,977	66	−	X
5	Abdominoplasty	30–40	−	−	−	3,7	912	2,465	72	X	0
6	Hemicolectomy	80–90	X	−	−	1, 8	564^*^	3,133	64	X	0
7	Hemicolectomy	70–80	X	−	−	1, 3	477	3,672	65	X	X
8	Abdominoplasty	60–70	−	−	−	1, 9	714	3,759	67	X	X
9	Whipple operation	70–80	X	−	−	2	1,294	6,469	71	X	0
10	Abdominoperineal resection	60–70	X	−	−	2	644	3,219	64	X	0
11	Hemicolectomy + liver resection	70–70	X	−	−	0	0	0	0	0	0
12	Hemicolectomy	50–60	X	X	−	1, 9	463^*^	2,439	71	X	−
13	Whipple operation	80–90	X	−	−	1, 4	259	1,852	60	−	−
14	Liver resection	60–70	X	−	−	0	0	0	0	0	0

* *Break not at waist: true value higher. X, yes; −, no; 0, no measurement*.

The full thickness skin grafts had a median tensile strength of 604 N/cm. It had a significantly higher tensile strength than both the biological and synthetic materials tested. Sutures through the skin also endured significantly higher loads than did sutures through the synthetic material. Comparison between the three test materials is shown in Table 2. Since the coated synthetic mesh is woven from monofilament polyester, it was not possible to calculate the cross-sectional area which is why tensile strengths are presented as N/cm width at the waistline. The weakest skin sample had a tensile strength of 156 N/cm, this was the only patient on long-term corticosteroid medication as well as the only patient suffering from diabetes. The mean thickness of the skin strips was 2 mm ranging from 1.3 to 3.7 mm. Since we were able to measure the thickness and thus cross-sectional area at the waistline in the skin preparations, tensile strength is also presented as N/cm^2^ in Table 1.

**Table 2:** Comparison between the different materials.

**Material**	**Number**	**Tensile-strength (N/cm)**	***P*-value**	**Number**	**Suture gave way (N)**	***P*-value**
Synthetic	6	40 (5)	0.0007	6	30 (5)	0.0007
Biologic	13	208 (36)	0.0005	7	63 (6, 1)	0.0687
Skin	12	604 (408)	REF	12	67 (8)	REF

*Median (IQR)*.

Not all specimens were tested with both 2–0 and 0 sutures due to insufficient sample material. When the amount of material was sufficient to allow for testing with both suture sizes, a mean value was calculated for the breaking point, shown in Table 1. In all but two cases, the skin sample was stronger than at least one of the sutures. When the suture did not break, the skin behaved elastically, and at the maximum force applied, the suture slowly tore a rift in the skin until it finally reached the edge and gave way.

## Discussion

Full-thickness skin has remarkable mechanical properties that make it potentially ideal as reinforcement material in parastomal hernia repair, and strength will not be a limitation in initial trials that are planned. This study contributes significantly to the information required before proceeding with clinical trials comparing full-thickness skin with currently available commercial materials as reinforcement in the repair of parastomal hernia.

The principle behind the measurement of tensile strength is straightforward and similar techniques have been used by others to measure the tensile strength of biologic and synthetic meshes, scar tissue, linea alba, and rectus sheath (21–23). Our test device is custom-made and has not been validated regarding reproducibility and precision. However, all samples were tested in the same setting giving it internal validity. Furthermore, measurement by others of tensile strength of Xenmatrix^TM^ mesh yielded results comparable to ours (23).

In this study, even the weakest skin sample had excellent tensile strength, way above the physiological tensile forces in the abdominal wall (estimated not to exceed 16 N/cm) (21). This implies that skin grafts can be knife-meshed without creating the risk for rupture. Meshing has been considered important in other forms of skin grafting to prevent the encapsulation of seromas and hematomas in this sensitive area; an event that would prevent proper healing (24, 25). There are also reasons to believe that knife-meshing may facilitate vascularization thereby improving graft ingrowth.

The present results also show that sutures through skin in many cases can endure higher loads than those through materials commonly used in the repair of abdominal hernias. This suggests that it is more important to carefully prepare and adapt the skin graft to ensure proper integration and ingrowth, since the strength of the reinforcement does not seem to depend on the strength of sutures (12, 26). Knowledge of the breaking point of sutures in graft material also gives us an indication of the number of sutures needed for a certain length of graft. Our results suggest that the closeness of suture material in skin grafts need not be that great, thereby minimizing the burden of foreign material, which is desirable even when using resorbable sutures.

The first scientific report of using skin as reinforcement material in modern surgery was published by *Otto Loewe* in 1913, shortly followed by a study by Rehn (26, 27). A review of *Rehn's* work was published by *Uihlein* and included the use of “cutis grafts” in a wide range of plastic surgery procedures and in at least 80 hernia repairs, where <10% of cases showed a poor result (12). In a cohort of 49 patients with incisional hernia, he observed six recurrences after 2–9 years of follow–up; a result that even today would be considered good. These early studies with relatively good results indicate that the method could well be an alternative today.

Another factor to be considered is the fate of the skin graft after an implantation. The tensile strength of skin recorded in this study represents the situation at the moment of implantation. We do not know how the strength changes under the influence of tissue remodeling and repair. The fate of the implanted skin grafts was considered by the early authors as well. Their main concern was the formation of epidermoid cysts, and they stressed the importance of suturing the “cutis graft” under tension. Whether epidermis should be removed or not was investigated by *Peer* in two studies (28, 29). He demonstrated that it is not possible to remove the epidermis without leaving epithelial remnants. These remnants formed microscopic, epithelium-lined cysts that de-epithelialized and were degraded within months. He also noted that if the epidermis is left intact, it will be degraded and totally absent in samples taken after 1 month. This led to the conclusion that removal of the epidermis is not necessary.

In a study by our research team, a specimen was taken from a full-thickness skin graft used in the repair of a giant ventral hernia, 3 years after implantation. Histological examination revealed complete transformation into fibrous tissue with total absence of skin adnexae (13).

This metamorphosis of implanted skin could be an important factor that makes it superior to currently available synthetic materials. Synthetic material does not change or break down after implantation, and even if synthetic mesh is incorporated into fascia, it still causes an inflammatory reaction and scarring. Despite this, parastomal hernias tend to relapse, which may be due to scar detraction and that the mesh fails to fuse with the stomal intestine. Autologous skin, on the other hand, does not provoke a foreign body reaction, and may have a greater ability to heal and fuse with the stomal intestine. This creates a bridge that could prevent recurrence between the reinforcement material and the stomal intestine. This was originally believed would be the case with the biological graft options available today. However, a systematic review made by *Slater* showed that recurrence rates are comparable to those using synthetic mesh (30).

A weakness of this study is the relatively small sample size. Even though differences between the groups are statistically significant, variation in the skin group suggests that the results should be interpreted with care. However, even the skin sample with the lowest tensile strength sustained forces around 10 times the physiologically forces believed to occur in the abdominal wall. This particular skin sample came from an elderly man operated for colon cancer, he also suffered from diabetes and had a history of long-term corticosteroid medication for polymyalgia rheumatica. Another comment could be that there are many other biological and synthetic materials apart from those used for comparison in this study. However, since the main objective of the study was to investigate whether full-thickness skin has tensile strength great enough to be used as a reinforcement in hernia repair, comparison with other materials is superfluous. Another aspect of mechanics not tested in this study, is elasticity. We do not know how elasticity affects the efficiency of reinforcement material in hernia repair. One could speculate that the risk for developing a pseudohernia is greater. On the other hand, compliance of the abdominal wall after repair could be greater, thereby reducing discomfort, and improving function.

A strength of this study is that it includes skin samples from a wide range of patients, more closely representing the diverse group suffering from parastomal hernia. The skin specimens included in the study showed considerable variation in thickness. This indicates that even though taken from approximately the same position on the body, skin specimens are likely to have substantial inter-individual variation. This must be considered if skin is to be used as reinforcement material in the future since variation in the thickness of synthetic materials is basically zero. Another strength of the study is that it compares skin specimens with materials used in hernia surgery today.

Our study shows that full-thickness skin grafts fully meet the tensile strength requirements for reinforcement of the abdominal wall and moreover are superior to other materials currently used in hernia repair. Based on the results on this study and a previously conducted animal study, a randomized controlled study comparing intraperitoneally placed full-thickness skin to conventional reinforcement materials in the repair of parastomal hernia has been initiated (registered at clinicaltrials.gov; ID NCT03667287) (15).

## Data Availability Statement

All datasets generated for this study are included in the article/supplementary material.

## Ethics Statement

The studies involving human participants were reviewed and approved by the regional board of ethics at Umeå University, Umeå (reference number: 2016/450-31). The patients/participants provided their written informed consent to participate in this study.

## Author Contributions

VH: study design, data collection, data analysis, and writing. OB: data collection, writing, and critical revision. UG and KS: conception and design, data analysis, writing, and critical revision.

### Conflict of Interest

The authors declare that the research was conducted in the absence of any commercial or financial relationships that could be construed as a potential conflict of interest.
